# The sequence and *de novo* assembly of hog deer genome

**DOI:** 10.1038/sdata.2018.305

**Published:** 2019-01-08

**Authors:** Wei Wang, Hui-Juan Yan, Shi-Yi Chen, Zhen-Zhen Li, Jun Yi, Li-Li Niu, Jia-Po Deng, Wei-Gang Chen, Yang Pu, Xianbo Jia, Yu Qu, Ang Chen, Yan Zhong, Xin-Ming Yu, Shuai Pang, Wan-Long Huang, Yue Han, Guang-Jian Liu, Jian-Qiu Yu

**Affiliations:** 1 Animal Breeding and Genetics Key Laboratory of Sichuan Province, Sichuan Animal Science Academy, Chengdu, China; 2 Chengdu Zoo, Chengdu, China; 3 Farm Animal Genetic Resources Exploration and Innovation Key Laboratory of Sichuan Province, Sichuan Agricultural University, Chengdu, China; 4 Novogene Bioinformatics Institute, Beijing, China

**Keywords:** Genome, Zoology

## Abstract

Hog deer (*Axis porcinus*) is a small deer species in family Cervidae and has been undergoing a serious and global decline during the past decades. Chengdu Zoo currently holds a captive population of hog deer with sufficient genetic diversity in China. We sequenced and *de novo* assembled its genome sequence in the present study. A total of six different insert-size libraries were sequenced and generated 395 Gb of clean data in total. With aid of the linked reads of 10X Genomics, genome sequence was assembled to 2.72 Gb in length (contig N50, 66.04 Kb; scaffold N50, 20.55 Mb), in which 94.5% of expected genes were detected. We comprehensively annotated 22,473 protein-coding genes, 37,019 tRNAs, and 1,058 Mb repeated sequences. The newly generated reference genome is expected to significantly contribute to comparative analysis of genome biology and evolution within family Cervidae.

## Background & Summary

There are 56 cervid species (family Cervidae) in the Red List of International Union for Conservation of Nature^1^ and form the second most diverse group among terrestrial artiodactyls^2^. Cervids are widely geographical distribution and show considerable variation on antler phenotype, body size and other morphologic features^3^. Therefore, they are the ideal materials for studying evolutionary dynamics of phenotypes and genetic adaptions to highly diverse environments^4^. With the development of high-throughput sequencing technologies^5^, genome sequences could be obtained in a more economical way and would largely facilitate biological researches in cervids. Although the draft genomes have been recently published for red deer (*Cervus elaphus*)^6^ and reindeer (*Rangifer tarandus*)^7^, a large number of cervid species remain to be sequenced.

**Figure 1:**
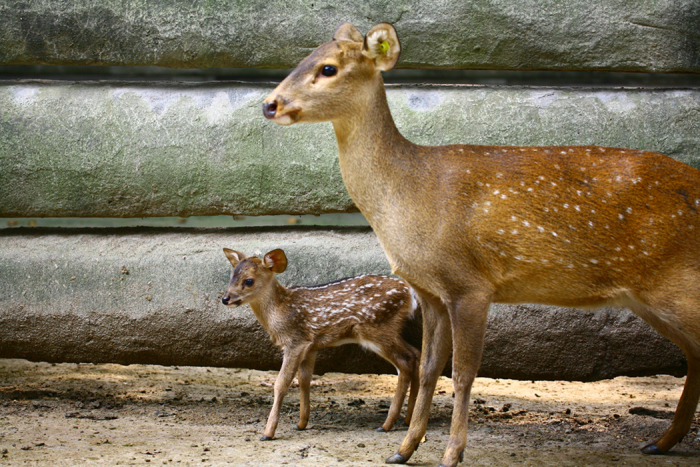
An adult female hog deer and its small baby in Chengdu Zoo.

Hog deer (*Axis porcinus*) is a small deer (30-50 kg adult weight) in Cervinae subfamily (Fig. 1) and mainly distributed in Pakistan, Nepal, India, Bangladesh, Burma, China, Thailand and Laos^8^. A specific feature of hog deer is that it has a narrow habitat in wet or moist tall grasslands. Recently, the wild hog deer has been recognized to globally decrease in population size and even to be almost completely eliminated in China^9,10^. Chengdu Zoo of Sichuan holds the largest captive population of hog deer in China, for which the genetic diversity has been successfully revealed by the genome-wide SNPs in our lab^11^. In the present study, we further sequenced and *de novo* assembled the genome of hog deer, which is expected to contribute to the comparative analysis of genome biology among cervid species.

## Methods

### Ethics statement

In the present study, blood sample was collected by veterinarian at annual health inspection and tissue samples for RNA extraction were obtained from the accidentally died individuals with fighting injury. The study design and all experimental methods were approved by Animal Care and Use Committee in Chengdu Zoo.

### Sample collection and construction of sequencing libraries

The blood was sampled from a healthy female hog deer at two years old. Genomic DNA was isolated using Qiagen DNA purification kit (Qiagen, Valencia, CA, USA). A total of six paired-end and mated-pair sequencing libraries with 250 bp, 350 bp, 450 bp, 2 Kb, 5 Kb and 10 Kb of insert sizes were constructed according to Illumina’s protocol (Illumina, San Diego, CA, USA). For insert sizes of 250 bp to 450 bp, 0.5 μg of genomic DNA was fragmented, end-paired, and ligated to adaptors, respectively. The ligated fragments were fractionated on agarose gels and purified by PCR amplification to produce sequencing libraries. For the mated-pair libraries with insert sizes of 2 Kb to 10 Kb, 120 μg of genomic DNA was circularized and digested. Furthermore, a 10X Genomics linked-read library was also constructed successfully according to protocol (10X Genomics, San Francisco, USA).

Six tissues including brain, heart, lung, liver, spleen and kidney were sampled for three hog deer. Subsequently, all 18 samples were subjected to RNA extraction using RNAiso Pure RNA Isolation Kit (TaKaRa, Japan), which was followed by DNaseI treatment. NanoVue Plus spectrophotometer (GE Healthcare, NJ, USA) was used to assess concentration and quality of the extracted RNAs. All RNA samples were sequenced by Illumina HiSeq X for generating paired-end reads in 150 bp which three same samples were pooled. All sequencing libraries constructed were detailed in Table 1.

### Sequencing and genome assembly

A total of 404 Gb sequencing data were generated from the Illumina’s paired-end sequencing. Read quality was analyzed using NGS QC Toolkit^12^ and the low-quality reads were discarded according to any one of the three criterions, including (1) reads containing adaptor sequences, (2) reads containing ambiguous bases more than 10% of total length, and (3) reads containing low-quality bases (Q-value < 5) more than 20% of total length. If any member of the paired reads was classified as low quality, both pairs were discarded. After filtering, 395.2 Gb clean bases were obtained for *de novo* assembly of genome. Also, 230.78 Gb clean bases, out of 236.7 Gb sequencing data, were obtained from 10X Genomics sequencing (Table 1).

SOAPdenovo2^13^ was employed for constructing contigs and scaffolds with the optimized parameters of -K 41 and -d 1 for the PREGRAPH step, -k 41 for MAP step, and -l 43 for SCAFF step, respectively. Briefly, contigs were first *de novo* assembled with short reads, against which all reads were aligned for constructing scaffolds with aid of the paired information of reads. Second, gaps were filled according to the paired information of reads. Third, these initially obtained scaffolds were further improved by incorporating the linked reads of 10X Genomics using Fragscaff^14^ with the parameters of -fs1 ‘-m 3000 -q 30’ -fs2 ‘-C 2’ -fs3 ‘-j 1.25 -u 2’. These processes finally yielded a draft genome of hog deer with a total length of 2.72 Gb, contig N50 of 66.04 Kb and scaffold N50 of 20.55 Mb (Table 2).

The completeness of genome assembly was assessed by three approaches as followed. The single copy orthologs set (BUSCO, version 2.0) were searched against the assembled genome of hog deer using BUSCO tool^15^, which revealed that 94.5% of the 843 expected genes are present in this assembly. Based on a core gene set involved in 248 evolutionarily conserved genes from six eukaryotic model organisms, the comparative analysis by CEGMA tool^16^ similarly revealed that 95.97% of these core genes have been successfully assembled. Finally, the Core Vertebrate Genes (CVG)^17^ was used as reference gene set to assess the completeness by gVolante tool (https://gvolante.riken.jp), which also showed that this assembly completely captured 216 core genes(92.70%).

### Annotation of genomic repeat sequences

Both homologous comparison and *ab initio* prediction were used to annotate the repeated sequences within hog deer genome. RepeatMasker and the associated RepeatProteinMask (-noLowSimple, -pvalue 0.0001, -engine wublast)^18^ were performed for homologous comparison by searching against Repbase database^19^. For *ab initio* prediction, LTR_FINDER^20^ (-C, -w 2), RepeatScout^21^ and RepeatModeler^22^ were first used for *de novo* constructing the candidate database of repetitive elements, by which the repeated sequences were annotated using RepeatMasker (-a, -nolow, -no_is, -norna). Tandem repeat was *ab initio* predicted using TRF (Match = 2, Mismatch = 7, Delta = 7, PM = 80, PI = 10, Minscore = 50, MaxPeriod = 2000, -d -h) tool^23^. According to these analyses, about 1,058 Mb repeat sequences were finally revealed, which accounted for 38.9% of the whole genome (Table 3).

### Annotation of gene structure

We employed three approaches for predicting the protein-coding genes within hog deer genome, including homologous comparison, *ab initio* prediction and RNA-seq based annotation. For homologous comparison, the reference protein sequences from Ensembl database (release 91) for five species of human (*Homo sapiens*), cattle (*Bos taurus*), water buffalo (*Bubalus bubalus*), sheep (*Ovis aries*) and bactrian camel (*Camelus bactrianus*) were aligned against hog deer genome using TBLASTN search with parameters of e-value 1e-5 in the “-F F” option^24^. After filtering low-quality records, all blast hits were concatenated. Sequence of each candidate gene was further extended upstream and downstream by 1,000 bp to represent the whole region of this gene, within which the gene structure was predicted using GeneWise tool^25^. RNA reads from six tissues were *de novo* assembled with Trinity^26^ (--normalize_reads, --full_cleanup, --min_glue 2, --min_kmer_cov 2, --KMER_SIZE 25) and the assembled sequences were aligned against hog deer genome using Program to Assemble Spliced Alignment (PASA), by which the effective alignments were assembled to gene structures^27^. We simultaneously employed five tools of Augustus^28^, GeneID^29^, GeneScan^30^, GlimmerHMM^31^ and SNAP^32^ for *ab initio* prediction, in which the parameters were computationally optimized by training a set of high-quality proteins that have been derived from the PASA gene models with default parameters. Simultaneously, RNA-seq reads were aligned to hog deer genome using TopHat with default parameters^33^, by which the mapped reads were assembled into gene models by Cufflinks^34^. According to these three approaches, the non-redundant reference gene set was finally generated using EvidenceModeler (EVM) tool^27^. In order to get the UTRs and alternative splicing variation information, we used PASA2 to update the gene models^27^. Finally, we successfully generated reference gene structures within hog deer genome, which is composed of 22,473 protein-coding genes (Table 4).

We also predicted gene structures of tRNAs, rRNAs and other non-coding RNAs (Table 5). A total of 37,019 tRNAs were predicted using t-RNAscan-SE tool (--evalue 1e-10)^35^. Because rRNA genes are highly evolutionarily conserved, we choose human rRNA sequence as references and then predicted 920 rRNA genes using Blast tool with default parameters^36^. Small nuclear and nucleolar RNAs were annotated using the infernal tool ^37^.

### Functional annotation of protein-coding genes

We functionally annotated the predicted proteins within hog deer genome according to homologous searches against three databases of SwissProt^38^, InterPro^39^ and KEGG pathway^40^. Of that, InterproScan tool^41^ in coordination with InterPro database^39^ were applied to predict protein function based on the conserved protein domains and functional sites. KEGG pathway and SwissProt database were mainly mapped by the constructed gene set to identify best match for each gene. Overall, 89.7%, 87.4%, 79.1% genes show positive hits in SwissProt, InterPro, and KEGG, respectively. In summary, a total of 20,994 genes (93.4%) were successfully annotated by function implications or the conserved functional motifs (Table 6).

### Code availability

The following bioinformatic tools and versions were used for generating all results as described in the main text:

1. NGS QC Toolkit, version 2.3.2, was used for quality filtering of reads: https://www.nipgr.res.in/ngsqctoolkit.html.
2. SOAPdenovo, version 2, was used for genome assembly: https://soap.genomics.org.cn/soapdenovo.html.
3. Fragscaff, version 140324, was used for scaffolding with 10X Genomics reads: https://sourceforge.net/projects/fragscaff/files/.
4. BUSCO, version 3.0.2, was used for assessing genome assembly completeness: https://busco.ezlab.org.
5. CEGMA, version 2.5, was used for assessing genome assembly completeness: https://korflab.ucdavis.edu/datasets/cegma/.
6. gVolante (an online tool), accessed at 11/2018, was used for assessing genome assembly completeness: https://gvolante.riken.jp/analysis.html.
7. RepeatMasker, version 4.0, was used for annotating repeated sequences: https://repeatmasker.org.
8. LTR_FINDER, version 1.0.5, was used to predict locations and structure of full-length LTR retrotransposons: https://github.com/xzhub/LTR_Finder.
9. TRF, version 4.07b, was used to *de novo* construct the candidate database: https://tandem.bu.edu/trf/trf.html.
10. RepeatScout, version 1.0.5, was used to *de novo* construct candidate database: https://bix.ucsd.edu/repeatscout/.
11. RepeatModeler, version 1.0.4, was used to *de novo* construct candidate database: https:// repeatmasker.org/RepeatModeler/.
12. blast, version 2.2.26, was used to align reads to genome sequences: https://blast.ncbi.nlm.nih.gov/Blast.cgi.
13. GeneWise, version 2.4.1, was used to predict gene structure: https://ebi.ac.uk/~birney/wise2/.
14. Trinity, version 2.0, was used for *de novo* genome assembly with RNA reads: https://github.com/trinityrnaseq/trinityrnaseq/wiki.
15. PASA, version 2.0.2, was used to model the gene structures: https://github.com/PASApipeline/PASApipeline/wiki.
16. Augustus, version 3.1, was used for *ab initio* prediction of gene structure: https://bioinf.uni-greifswald.de/augustus/.
17. GeneID, version 1.4, was used for *ab initio* prediction of gene structure: https://genome.crg.es/software/geneid/.
18. GeneScan, version 1.0, was used for *ab initio* prediction of gene structure: https://genes.mit.edu/GENSCAN.html.
19. GlimmerHMM, version 3.0.4, was used for *ab initio* prediction of gene structure: https://ccb.jhu.edu/software/glimmerhmm/.
20. SNAP, version 2013-02-16, was used for *ab initio* prediction of gene structure: https://snap.cs.berkeley.edu.
21. TopHat, version 2.09, was used to align RNA reads to genome sequences: https://ccb.jhu.edu/software/tophat/index.shtml.
22. Cufflinks, version 2.2.1, was used to assemble RNA reads into gene models: https://cole-trapnell-lab.github.io/cufflinks/cuffdiff/index.html.
23. EVM, version 1.1.1, was used to combine *ab initio* gene predictions and generate the consensus gene structures: https://evidencemodeler.github.io.
24. t-RNAscan-SE, version 1.4, was used to search tRNA: https://lowelab.ucsc.edu/tRNAscan-SE/.
25. infernal, version 1.1rc4, was used to predict miRNA and snRNA: https://eddylab.org/infernal/.

## Data Records

A total of 12 sequencing runs of DNA-seq (SRR7410909-17, SRR7410919-21) and six runs of RNA-seq (SRX4282445-49, SRX4282453) were obtained and deposited to NCBI Sequence Read Archive (SRA) (Data Citation 1). The assembled draft genome has been deposited at GenBank (Data Citation 2). The annotation results of repeated sequences, gene structure and functional prediction were deposited in Figshare database (Data Citation 3).

## Technical Validation

### RNA integrity

In prior to constructing RNA-seq libraries, the concentration and quality of total RNA were evaluated using Agilent 2100 Bioanalyser (Agilent, Santa Clara, USA). Three metrics, including total amount, RNA integrity and rRNA ratio, were used to estimate the content, quality and degradation level of RNA samples. In this study, only total RNAs with a total amount ≥ 10 μg, RNA integrity number ≥ 8, and rRNA ratio ≥ 1.5 were finally subjected to construct the sequencing library.

### Quality filtering of raw reads

The initially generated raw sequencing reads were evaluated in terms of the average quality score at each position, GC content distribution, quality distribution, base composition, and other metrics. Furthermore, the sequencing reads with low quality were also filtered out before the genome assembly and annotation of gene structure.

## Additional information

**How to cite this article**: Wang, W. *et al*. The sequence and *de novo* assembly of hog deer (Axis porcinus) genome. *Sci. Data*. 6:180305 doi: 10.1038/sdata.2018.305 (2019).

**Publisher’s note**: Springer Nature remains neutral with regard to jurisdictional claims in published maps and institutional affiliations.

## Supplementary Material



## Figures and Tables

**Table 1:** Library information and sequencing results.

Types	Libraries	Insert sizes	Raw data (Gb)	Clean data (Gb)
Genomic DNA sequencing	DES01754	250 bp	102.4	101.68
	DES01765	350 bp	74.1	73.59
	DES01755	450 bp	72.3	71.62
	DEL01229	2 Kb	31.5	30.56
	DEL01226	2 Kb	34.4	34.40
	DEL01227	5 Kb	33.3	31.36
	DEL01230	5 Kb	27.2	26.78
	DEL01228	10 Kb	13.7	12.38
	DEL01231	10 Kb	14.65	12.83
	KD17051609	10X Genomics	236.7	230.78
RNA sequencing	RRA59894-S	250 bp	7.2	7.12
	RRA59895-S	250 bp	8.8	8.66
	RRA59896-S	250 bp	8.3	8.16
	RRA59897-S	250 bp	10.0	9.86
	RRA59898-S	250 bp	9.1	9.00
	RRA59899-S	250 bp	10.0	9.94

**Table 2:** The *de novo* assembled genome of hog deer.

	Length, bp		Number	
	Contigs	Scaffolds	Contigs	Scaffolds
Total	2,679,167,314	2,719,585,391	544,656	463,740
Max	794,078	91,389,359	—	—
N50	66,035	20,551,061	11,195	40
N90	9,852	1,790,557	47,200	170

**Table 3:** Annotation of repeated sequences.

Tools	Repeat Size (bp)	% of genome
RepeatMasker	1,016,366,209	37.37
RepeatProteinMask	439,972,572	16.10
TRF	42,982,131	1.58
Total	1,057,944,353	38.90

**Table 4:** Prediction of protein-coding genes.

Methods / Tools		Gene number	Exons per gene	Average length (bp)			
Gene	CDS			Exon	Intron		
Homologous comparison	*H. sapiens*	34,654	5.21	15,443.28	1,052.42	202.17	3,421.74
	*B. taurus*	26,310	5.55	16,413.01	1,154.44	207.95	3,352.45
	*B. bubalus*	71,084	3.64	8,528.97	779.38	214.37	2,940.32
	*O. aries*	73,148	3.48	8,194.63	732.05	210.60	3,013.96
	*C. bactrianus*	25,194	6.60	20,193.20	1,269.57	192.35	3,378.97
RNA-seq		81,311	8.05	37,959.37	3,869.12	480.89	4,838.45
*Ab initio* prediction	Augustus	36,909	4.67	14,638.62	1,002.89	214.88	3,718.34
	GlimmerHMM	557,641	2.41	4,014.61	424.63	176.26	2,547.72
	SNAP	128,744	3.53	25,890.73	530.45	150.38	10,034.10
	GenID	286,917	1.64	4,298.66	190.45	115.91	6,388.70
	GeneScan	71,999	5.48	24,967.54	920.23	168.05	5,372.64
EVM		44,470	3.92	16,031.05	957.78	194.69	3,845.72
Final set		22,473	8.61	34,536.59	1,449.48	172.73	4,476.40

**Table 5:** Annotation of non-coding RNA genes.

Type		Copy	Average length (bp)	Total length (bp)	% of genome
rRNA	miRNA	17,289	97.54	1,686,371	0.06
	tRNA	37,019	72.90	2,698,717	0.10
	rRNA	920	97.94	90,101	0.01
	18 S	51	131.27	6,695	0.00
	28 S	250	143.38	35,844	0.00
	5.8 S	4	81.25	325	0.00
	5 S	615	76.81	47,237	0.00
snRNA	snRNA	4119	102.84	423,601	0.02
	CD-box	501	92.24	46,212	0.00
	HACA-box	607	132.91	80,680	0.00
	Splicing	2925	97.20	284,299	0.01

**Table 6:** Functional annotation of the predicted protein-coding genes.

Methods for annotation	Number	Percent (%)
Swissprot	20,162	89.7
InterPro	19,650	87.4
KEGG	17,783	79.1
NR	20,957	93.3
Annotated	20,994	93.4
Unannotated	1,479	6.6

## References

[d1] NCBI Sequence Read Archive 2018 SRP151090

[d2] GenBank 2018 QQTR00000000

[d3] Figshare Chen S. Y. 2018 10.6084/m9.figshare.7176116.v1

